# Gait improvement after levofloxacin administration in a progressive supranuclear palsy patient

**DOI:** 10.1016/j.prdoa.2020.100080

**Published:** 2020-11-29

**Authors:** Masanori Kurihara, Kenta Orimo, Tatsuo Mano, Ryoji Miyano, Aya Kamisawa Sato, Kenichiro Sato, Ryoko Ihara, Toshihiro Hayashi, Tatsushi Toda

**Affiliations:** a Department of Neurology, The University of Tokyo, 7-3-1 Hongo, Bunkyo-ku, Tokyo 113-8655, Japan; b Unit for Early and Exploratory Clinical Development, The University of Tokyo Hospital, 7-3-1 Hongo, Bunkyo-ku, Tokyo 113-8655, Japan

**Keywords:** Parkinsonian disorders, Progressive supranuclear palsy, Levofloxacin, Fluoroquinolones

## 1 Introduction

Progressive supranuclear palsy (PSP) is a neurodegenerative disease that presents with various clinical symptoms. Although Richardson’s syndrome is the most common, other presentations include parkinsonism (PSP-P) and progressive gait freezing [1]. In contrast to Parkinson’s disease (PD), PSP has few treatment options and the response to anti-parkinsonian drugs is limited.

Herein, we report a patient with PSP whose gait was consistently improved after levofloxacin administration.

## 2 Case report

A 55-year-old man presented with bradykinesia and right hemi-tremor. Although the patient responded partially to levodopa and dopamine agonists, the symptoms progressed and stuttering speech, vertical gaze palsy, postural instability, and gait freezing appeared. ^123^I-Ioflupane SPECT showed decreased accumulation in the bilateral striatum, including the caudate nucleus. ^123^I-MIBG myocardial scintigraphy showed normal results. On the brain MRI, midbrain atrophy was indefinite, and bilaterally, mild atrophy of the globus pallidus and thalamus was noted. We diagnosed him as having probable PSP-P [1]. Since further adjustment of drug dosages showed no improvement, or partial improvement of rigidity but worsening of gait festination and falls, the patient was administered daily doses of levodopa/carbidopa 500 mg, ropinirole 2 mg, and amantadine 100 mg. However, the patient still showed severe gait freezing and frequent falls.

At the age of 67 years, he was prescribed levofloxacin and loxoprofen (non-steroidal anti-inflammatory drug [NSAID]) for pharyngitis by a primary care physician. Surprisingly, two days later, the patient experienced improvement in his gait. He showed better posture, less gait freezing, and could walk approximately 20 steps without assistance. In contrast, normally the patient could only walk a few steps. After five days, levofloxacin administration was stopped, and his gait returned to the baseline level the next day. The patient was hospitalized for medical evaluation.

On admission, physical examination and blood tests were negative for any infection. The patient presented with a stuttering voice, upward gaze palsy, gait freezing, postural instability, and postural/resting tremor. He took 86 s to complete the 5 m Timed Up and Go test (TUG) using a four-wheeled walker. His PSP rating scale (PSPRS) was 42/100. Five days after admission, when considering the possibility of using levofloxacin for his neurological symptoms, he coincidentally suffered from diverticulitis. We started levofloxacin 500 mg once daily for the diverticulitis and evaluated whether his gait improved again. Two days later, the patient showed improved gait freezing and shuffling gait. The patient completed the TUG test in 61 s on day 3, 50 s on day 7, and 63 s on day 14, after starting levofloxacin. His PSPRS on day 7 was 40/100. After the discontinuation of levofloxacin, the patient showed worsened gait freezing and shuffling gait and took 72 s to complete the TUG. His PSPRS returned to 42/100, and there were no signs of recurrence of the infection.

**Fig. 1:**
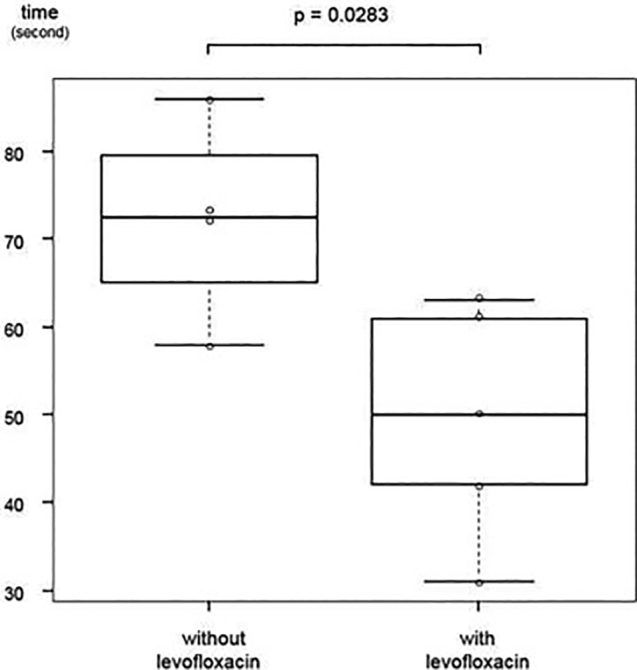
The results of the 5 m Timed Up and Go tests (TUG) of the patient during hospitalization and outpatient visits. Circles represent each result (n = 4 without levofloxacin, n = 5 with levofloxacin), and the box-and-whisker plots are shown. Significance of difference between TUG results without levofloxacin and with levofloxacin was determined using the Welch test. A p-value of less than 0.05 was considered statistically significant. TUG results were significantly better while on levofloxacin.

The patient and his wife requested to continue levofloxacin for symptomatic management. After ethical approval, the patient was prescribed levofloxacin for 1 week, followed by an off-period of 2–3 weeks, for the subsequent 5 months. The TUG test was repeated at each follow-up visit. At each levofloxacin administration, the patient and his wife noticed improved activation and gait, and the time to complete the TUG test was significantly shorter while on levofloxacin (Fig. 1).

## 3 Discussion

Our patient consistently showed gait improvement following levofloxacin administration.

We were only able to find a single published case report of parkinsonian symptom improvement after levofloxacin administration. El Ayoubi et al. reported a patient with PD who repeatedly showed improvement of bradykinesia and gait instability, 2 days after levofloxacin, with symptoms returning to baseline level 2 days after discontinuation of levofloxacin [2]. Since drug interaction was less likely, the authors hypothesized that the direct central nervous system (CNS) effect of levofloxacin, including its anti-GABAergic effect, which is known to be related to its CNS side effects, may have contributed to the patient’s symptomatic improvement [2]. This mechanism of action has also been considered in other reports of levofloxacin administration for improvement of neuropsychiatric conditions, such as apathy [3]. The same authors also discussed the possibility that NMDA receptor activation contributed to the improvement of apathy [3]. Since NMDA receptor activation has been reported to worsen parkinsonism [4], [5], improvement of parkinsonian gait, especially gait freezing, in our patient would be more likely due to the anti-GABAergic effect of levofloxacin on excess GABAergic activity within the basal ganglia circuit. However, the NMDA agonist effect of levofloxacin may have improved his alertness or emotional state and subsequently his gait. In the current case, the patient and his wife reported that the first improvement while on levofloxacin plus NSAID was most dramatic; this drug combination is known to augment the anti-GABAergic effect of levofloxacin and increase the risk of epilepsy. Although we cannot completely rule out the influence of these drugs on the intestinal microflora and the absorption of other drugs, this episode may also suggest that the anti-GABAergic effect of the drug is involved in gait improvement.

The role of GABAergic neurons in movement control and parkinsonian symptoms has long been investigated. Akinesia and gait freezing are considered to be related to increased GABAergic output from the basal ganglia to the mesencephalic locomotor center [6]. In PD, there are reports that an intravenous GABA antagonist, flumazenil, may improve akinesia/bradykinesia [7], while others reported improvement after administration of a GABA “agonist” in PD and PSP [8], [9]. Since there are multiple GABAergic pathways in the basal ganglia system with different effects on movement control, the final effect of global GABA modulation may depend on the remaining neurons and balance of the basal ganglia system in each patient. Our patient showed severe gait freezing and his MRI showed atrophy of the bilateral globus pallidi and thalami, but the midbrain volume was preserved. We hypothesize that levofloxacin mainly antagonizes the preserved and activated GABA receptors of the mesencephalic locomotor center neurons in the midbrain, thereby contributing to the improvement of gait freezing in our patient.

Although this case suggests that levofloxacin may improve symptoms in some patients with PSP, caution is necessary, since side effects of levofloxacin include epilepsy, encephalopathy, myoclonus, and emergence of antibiotic-resistant bacteria.

## 4 Conclusion

Levofloxacin may improve symptoms in some patients with PSP.

## 5 Ethics statement

All procedures followed were in line with the journal's ethics policy. Approval was obtained from the ethical review committee of the University of Tokyo Hospital (CL2017052), and informed written consent was obtained from the patient.

## Funding

This research was partially supported by the 10.13039/501100001691Japan Society for the Promotion of Science (JSPS) KAKENHI Grant Numbers JP19J10924 (to M.K.)

## Declaration of Competing Interest

The authors declare that they have no known competing financial interests or personal relationships that could have appeared to influence the work reported in this paper.

## References

[1] Höglinger G.U., Respondek G., Stamelou M. (2017). Clinical diagnosis of progressive supranuclear palsy: the movement disorder society criteria. Mov. Disord..

[2] El Ayoubi N., Sawaya R. (2016). Does levofloxacin improve parkinsonian features or is the improvement only coincidental?. Clin. Neuropharmacol..

[3] Armstrong M.J., Fox S.H., Marras C. (2012). Improvement of apathy after levofloxacin treatment: an N-of-1 study. Neurologist.

[4] Duty S. (2012). Targeting glutamate receptors to tackle the pathogenesis, clinical symptoms and levodopa-induced dyskinesia associated with Parkinson's disease. CNS Drugs.

[5] Rajrut A.H., Uitti R.J., Fenton M.E., George D. (1997). Amantadine effectiveness in multiple system atrophy and progressive supranuclear palsy. Parkinsonism Relat. Disord..

[6] Snijders A.H., Takakusaki K., Debu B. (2016). Physiology of freezing of gait. Ann. Neurol..

[7] Ondo W.G., Silay Y.S. (2006). Intravenous flumazenil for Parkinson's disease: a single dose, double blind, placebo controlled, cross-over trial. Mov. Disord..

[8] Daniele A., Albanese A., Gainotti G., Gregori B., Bartolomeo P. (1997). Zolpidem in Parkinson's disease. Lancet.

[9] Daniele A., Moro E., Bentivoglio A.R. (1999). Zolpidem in progressive supranuclear palsy. N. Engl. J. Med..

